# The Effect of Urinary Polycyclic Aromatic Hydrocarbon Metabolites on Lipid Profiles: Does Oxidative Stress Play a Crucial Mediation Role?

**DOI:** 10.3390/toxics12100748

**Published:** 2024-10-15

**Authors:** Yuting Wang, Jia Xu, Liujie Yang, Nan Zhang, Liwen Zhang, Bin Han

**Affiliations:** 1 Department of Occupational and Environmental Health, School of Public Health, Tianjin Medical University, Tianjin 300070, China; wangyuting@tmu.edu.cn (Y.W.); yangliujie@tmu.edu.cn (L.Y.); 2 State Key Laboratory of Environmental Criteria and Risk Assessment, Chinese Research Academy of Environmental Sciences, Beijing 100012, China; xu.jia@craes.org.cn (J.X.); zhangnan01@craes.org.cn (N.Z.)

**Keywords:** polycyclic aromatic hydrocarbon metabolites, 8-OHdG, blood lipid profiles, oxidative stress, mediation effect

## Abstract

Urinary polycyclic aromatic hydrocarbon (PAH) metabolites are associated with oxidative stress; however, epidemiological studies have not reported the impacts of these urinary PAH metabolites on blood lipid levels. This study investigated the relationship between urinary PAH metabolites, urinary 8-hydroxy-2′-deoxyguanosine (8-OHdG), and blood lipid profiles. A total of 109 elderly volunteers were recruited with complete datasets for analysis. Blood and morning urine samples were collected in the winter of 2011. The PAH metabolites, creatinine, and 8-OHdG levels in urine samples were analyzed using Gas Chromatography–Mass Spectrometry, spectrophotometry, and an ELISA kit, respectively. The blood lipid profiles were analyzed using an automatic biochemical analyzer. The relationship between lipid profiles and 8-OHdG was assessed using a two-independent sample nonparametric test, categorized by gender, smoking, and alcohol consumption status. After normalizing the concentration values, a general linear regression model was employed to examine the correlations between PAH metabolites, 8-OHdG, and lipid profiles. A mediation model was developed to investigate the mediating effect of 8-OHdG on the relationship between PAH metabolites and lipid profiles. The median of eight PAH metabolite concentrations in urine samples ranged from 1 to 10 μmol/mol creatinine (Cr). Significant differences in lipid profiles were observed across genders. However, no significant differences were found in smoking or alcohol consumption status for both genders. Linear regression analysis revealed that an increase in the logarithmic concentration of 2-hydroxynaphthalene (2-OHNap), 9-hydroxyfluorene (9-OHFlu), 3-hydroxyfluorene (3-OHFlu), 2-hydroxyfluorene (2-OHFlu), 1-hydroxypyrene (1-OHPyr), and 6-hydroxychrysene (6-OHChr) was associated with an increase in urinary 8-OHdG levels, after adjusting for BMI and age. Specifically, 1-hydroxynaphthalene (1-OHNap) and 1-OHPyr correlated negatively with apolipoprotein A1 (Apo A1). Conversely, 1-OHPyr was positively correlated with low-density lipoprotein cholesterol (LDL-C). In addition, b,c-dihydroxyphenanthrene (2-OHBcPhe) was positively associated with apolipoprotein B (Apo B). Notably, 8-OHdG did not exhibit a significant correlation with lipid profiles. The mediating effect of 8-OHdG on the relationship between hydroxylated PAHs and lipid profiles was not statistically significant. However, the indirect effects of hydroxylated PAHs on blood lipids were statistically substantial, specifically for 1-OHNap to Apo A1 (−0.025, 95% CI: −0.041, −0.009), 1-OHPyr to LDL-C (0.107, 95% CI: 0.011, 0.203), and 2-OHBcPhe to Apo B (0.070, 95% CI: 0.005, 0.135). This study suggests that an increase in urinary PAH metabolites may elevate the levels of urinary 8-OHdG and influence blood lipid profiles. However, no direct relationship was found between 8-OHdG and lipid profiles. The mediation analysis indicated that the effects of PAH metabolites on lipid changes may operate through pathways other than oxidative stress.

## 1. Introduction

Polycyclic aromatic hydrocarbons (PAHs) are a class of air pollutants characterized by two or more fused benzene rings, which can be arranged in linear, angular, or branched structures [1,2]. PAHs are recognized as hazardous organic pollutants due to their bioaccumulation propensity and environmental degradation resistance. Hydroxylated polycyclic aromatic hydrocarbons (OH-PAHs), metabolites derived from internal PAH exposure, have been closely linked to various adverse health outcomes. Numerous studies have demonstrated associations between OH-PAHs and reproductive toxicity [3], neurotoxicity [4], and inflammatory responses [5].

8-Hydroxy-2-deoxyguanosine (8-OHdG) is a modified nucleobase that forms in DNA following oxidative damage by hydroxyl radicals. It serves as a widely accepted biomarker for DNA damage [6,7,8]. Research has indicated that there is a correlation between the urinary levels of 8-OHdG and PAH metabolites. For instance, a study involving children found that the urinary levels of 1-hydroxypyrene (1-OHPyr) were positively correlated with 8-OHdG levels [9]. A statistically significant Pearson correlation was also observed between 1-OHPyr and 8-OHdG among Swedish ship engine room workers [10]. Furthermore, a study investigating the impact of traffic-related air pollution on DNA damage reported that a 75% increase in urinary 1-hydroxypyrene-glucuronide (1-OHPG) levels was associated with a 7.6% increase in urinary 8-OHdG levels [11]. Among these metabolites, 1-OHPyr is particularly valued as a biomarker for internal exposure due to its high specificity. Consequently, increasing concentrations of PAH metabolites might be associated with elevated levels of 8-OHdG, leading to enhanced DNA damage.

Recent studies have increasingly linked pollutants, including particulate matter (PM) and PAHs associated with PM, to cardiovascular diseases [12,13,14]. Lipid profiles, comprising total cholesterol (TC), triglycerides (TG), high-density lipoprotein (HDL), and low-density lipoprotein (LDL), apolipoprotein A1 (Apo A1), apolipoprotein B (Apo B), and also the ratio of Apo B to Apo A1, etc., are recognized as vital diagnostic indicators for cardiovascular disease [15,16,17]. However, research investigating PAH exposure and lipid profile associations remains limited. Most studies have concentrated on the effects of PAHs on lipid oxidative damage, often assessed using malondialdehyde (MDA) as a biomarker. For example, a survey conducted among individuals living near e-waste recycling facilities reported a significant positive correlation between urinary 1-OHPyr levels and MDA (r = 0.284, *p* < 0.01) [18]. Another investigation, which examined the migration between two cities, found a significant association between the sum of 12 OH-PAHs and MDA levels (*p* < 0.01), with variations observed between the two locations [19]. These studies suggest that OH-PAHs can influence MDA levels, while research focusing specifically on lipid profiles is scarce. Recent experimental studies have suggested that exposure to PAHs can significantly impair lipid metabolism and induce blood lipid elevations in mice [20,21,22].

This study investigated the relationship between PAH metabolites, 8-OHdG, and lipid profiles. Furthermore, we sought to determine whether oxidative stress mediates the impact of urinary PAH metabolites on blood lipid profiles. By elucidating these associations, this research may contribute to a better understanding of the health implications of PAH exposure and its potential role in cardiovascular disease risk.

## 2. Materials and Methods

### 2.1. Description of Demographic Information 

**Figure 1:**
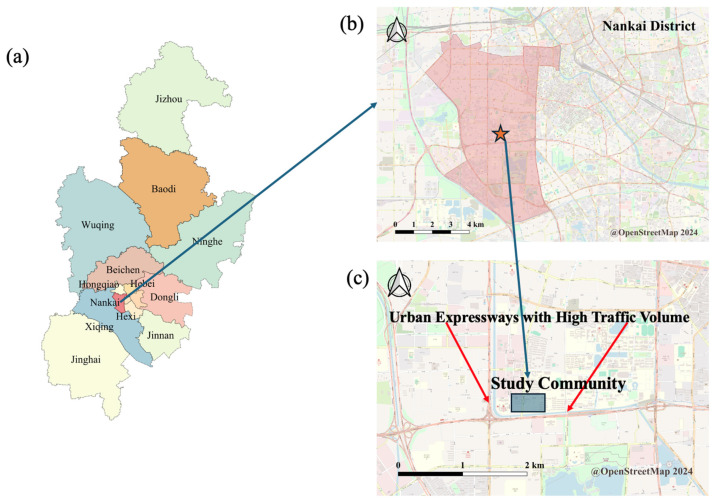
Location of the community in this study ((**a**) Tianjin; (**b**) the district where the study community is located; (**c**) the study community).

One hundred and twelve elderly volunteers over 60 were recruited from 2000 individuals in two communities within the Nankai District of Tianjin (Figure 1). Ultimately, 109 participants (46 males and 63 females) provided complete data. Each participant underwent a physical examination from 30 November to 12 December 2011. Morning urine and venous blood samples were collected from all participants for subsequent analyses. Throughout the study, participants maintained their regular dietary habits and lifestyle. Additionally, a questionnaire was administered by trained investigators to gather general demographic information, as well as data on smoking and alcohol consumption.

The study was conducted in accordance with the Declaration of Helsinki and approved by the Ethics Committee of Tianjin Medical University. Before participating, all participants provided informed consent.

### 2.2. Chemical Analyses on Urinary PAH Metabolites

The target metabolites in this study included 1-hydroxynaphthalene (1-OHNap), 2-hydroxynaphthalene (2-OHNap), 9-hydroxyfluorene (9-OHFlu), 3-hydroxyfluorene (3-OHFlu), 2-hydroxyfluorene (2-OHFlu), 1-hydroxypyrene (1-OHPyr), 6-hydroxychrysene (6-OHChr), and b,c-dihydrodiolsphenanthrene (2-OHBcPhe). The analysis procedure was based on the protocol by Li et al. [23] and Ma et al. [24].

Morning samples were collected on the day of the physical examination. These samples were aliquoted into 10 mL centrifuge tubes and stored in refrigerators at −20 °C for subsequent analysis. Before analysis, urine samples were thawed at room temperature. A 5 mL aliquot of urine was transferred to a 10 mL tube. The pH was adjusted to 5.0 using hydrochloric acid. Subsequently, 2.5 mL of 5 mol/L acetic acid, a sodium acetate buffer, and 20 µL of β-glucuronidase aryl sulfatase were added. The samples were hydrolyzed for 4 h at 37 °C without light, followed by centrifugation at 2000 rpm. Five milliliters of the hydrolyzed urine were then mixed with hexane in a 10 mL tube, agitated for 20 min, and allowed to stand to separate the supernatant. This process was repeated three times, and the supernatants were combined. The samples were evaporated to dryness, after which 200 µL of the derivatization reagent Bis(trimethylsilyl) trifluoroacetamide (BSTFA) was added, and the mixture was derivatized for 20 min at 60 °C in the absence of light. The resultant derivative solution was transferred to solid-phase extraction (SPE) cartridges for cleanup. PAH metabolites in urine were subsequently analyzed using a GC-MS system (6890N-5973i, Agilent, Santa Clara, CA, USA). The initial temperature of the GC oven was set at 60 °C for 3 min, after which it was increased at a rate of 40 °C/min to 150 °C and maintained for 3 min, followed by a further increase to 320 °C in 10 min, where it was held for 2 min. High-purity helium was used as the carrier gas at a 1.0 mL/min flow rate. The scanning mode was used for ion monitoring with the following mass-to-charge ratios: 216 amu and 201 amu (1-OHNap, 2-OHNap); 254 amu and 239 amu (2-OHFlu, 3-OHFlu, 9-OHFlu); 266 amu and 251 amu (1-OHPhe, 2-OHPhe, 3-OHPhe, 4-OHPhe, 9-OHPhe); 316 amu and 301 amu (2-OHBcPhe, 6-OHChr); and 299 amu and 275 amu (1-OHPyr). Detailed information on the analysis is introduced in Text S1 and Table S1. Figure S1 displays the total ion chromatogram of a mixed standard sample of urinary PAH metabolites.

### 2.3. Analyses of the Biomarkers

#### 2.3.1. Urinary 8-OHdG Analysis

After being thawed at room temperature, a 1 mL aliquot of urine was placed in a centrifuge tube and centrifuged at 3200× *g* for 20 min. The concentration of 8-OHdG in the urine was determined using an ELISA kit (JaICA, Koishikawa, Japan).

#### 2.3.2. Correction of Urinary PAH Metabolites and 8-OHdG by Creatinine

Creatinine concentrations in urine samples were measured using spectrophotometry (U-3900, Hitachi, Tokyo, Japan) with picronitric acid as a chromogenic agent. Creatinine levels subsequently adjusted the concentrations of PAH metabolites and 8-OHdG to account for individual variability in urine concentration.

#### 2.3.3. Lipid Profiles Analysis

Venous blood samples were collected during the physical examination. A total of 5 mL of blood was drawn into an anticoagulant tube containing EDTA-K2 and centrifuged at 3200 rpm for 10 min. The separated plasma was stored at −80 °C for subsequent analysis. The lipid profiles, including total cholesterol (TC), triglycerides (TG), low-density lipoprotein (LDL), and high-density lipoprotein (HDL), were measured using a chemical analyzer (AU5800, Beckman Coulter, Brea, CA, USA) according to the manufacturer’s protocols.

### 2.4. Statistical Analysis

**Figure 2:**
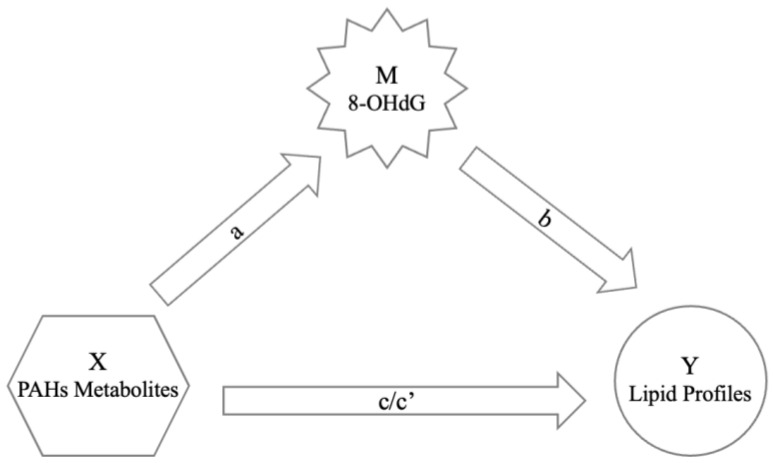
The mediation model: 8-OHdG (M) as the means of mediation between OH-PAHs (X) and lipid profiles (Y). (a denotes the effects of OH-PAHs on 8-OHdG; b denotes the effects of 8-OHdG on lipid profiles; c denotes the total effect of OH-PAHs on lipid profiles; c’ denotes the direct effect of OH-PAHs on lipid profiles after controlling for 8-OHdG).

Normal distribution data were presented as mean ± standard deviation, while non-normal distribution data were presented as geometric mean and quartiles. We assessed whether lipid profile and 8-OHdG showed statistically significant differences among elderly individuals according to gender, smoking status, and alcohol status through a two-independent nonparametric test. As all data showed a non-normal distribution, we normalized the concentration values by taking the logarithm of the concentrations. A general linear regression model analyzed the associations between PAH metabolites, 8OHdG, and lipid profiles. Two models were developed in this study: one was a base model without any adjustment (**Model 1**); the other one was adjusted by age and BMI (**Model 2**). We used bootstrapping to analyze whether the effect of OH-PAHs on lipid profiles is mediated through 8-OHdG [25,26]. Figure 2 shows the assumption of this study that 8-OHdG is a mediator (M) of the relationship between PAH metabolites (X, independent variables) and lipid profiles (Y, dependent variables). In bootstrap sampling, the number of samples was set to 5000, and the bias-corrected method was used. The confidence level was 95% in the setting. The total, direct, and indirect effects were estimated using ordinary least squares (OLS) regression analyses separately for lipid profiles. The total effect showed no mediator variable (8-OHdG), while the direct effect indicated the effect of OH-PAHs on lipid profiles when controlling the mediator variable. The indirect effect showed the effect of the mediation path. The total effect “c” of X on Y can be decomposed into direct effect “c’” and indirect effect “a and b”. The “a” was the effect of X on M. The “b” was the effect of M on Y when controlling X. The “a × b” was the effect of mediation. All tests of significance were two-sided. Statistical significance was defined as *p* < 0.05. All statistics were performed using IBM SPSS Statistics for Windows (IBM Corporation, New York, NY, USA, 2013 version) and the PROCESS macro for SPSS (Hayes 2013).

## 3. Results

This study recruited 109 participants, including 46 men and 63 women. The statistical characteristics of these participants are shown in Table 1. The average age of the male participants was 68.78 ± 7.00 years, while it was 66.79 ± 5.99 years for female participants. The baseline data of body mass index (BMI), smoking status, and alcohol consumption are also summarized in Table 1. Significant differences were observed in smoking and alcohol status between genders.

**Figure 3:**
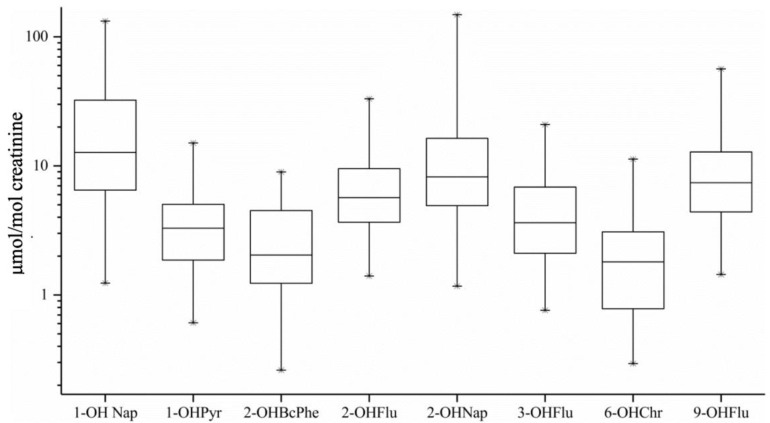
Concentrations of the urinary OH-PAHs corrected by creatinine.

Figure 3 illustrates the concentrations of various polycyclic aromatic hydrocarbon (PAH) metabolites in urine, adjusted for creatinine levels. The median concentrations of these PAH metabolites ranged from approximately 1 to 10 μmol/mol Cr. The urinary concentrations of hydroxylated PAHs (OH-PAHs) were ranked in a descending order, as follows: 1-OHNap > 2-OHNap > 9-OHFlu > 2-OHFlu > 3-OHFlu > 1-OHPyr > 2-OHBcPhe > 6-OHChr. Notably, the low-ring compounds, Nap and Flu, were the significant components of PAH metabolites in urine, accounting for 56% and 19% of the total, respectively.

The lipid profiles assessed total cholesterol (TC), triglycerides (TG), high-density lipoprotein cholesterol (HDL-C), low-density lipoprotein cholesterol (LDL-C), apolipoprotein A1 (Apo A1), apolipoprotein B (Apo B), and the ratio of Apo B to Apo A1. To evaluate potential differences in lipid profiles and 8-hydroxy-2′-deoxyguanosine (8-OHdG) in genders, smoking and alcohol status, a two-independent nonparametric test was conducted. As shown in Table 2, the concentrations of TC, HDL-C, LDL-C, Apo A1, and Apo B displayed significant differences between genders, with the median concentrations in women being higher than those in men. However, no significant differences were observed in lipid profiles based on smoking or alcohol status.

Given that the distribution of all substances was non-normal, we applied logarithmic transformation for normalization. Following normalization, a general linear regression model was employed to investigate the correlations between OH-PAHs, 8-OHdG, and lipid profiles. The results presented in Table 3 indicate that several PAH metabolites were associated with 8-OHdG levels. Specifically, linear regression analysis revealed that increases in the logarithmic concentrations of 2-OHNap, 9-OHFlu, 3-OHFlu, 2-OHFlu, 1-OHPyr, and 6-OHChr corresponded to increases of 0.305, 0.420, 0.456, 0.467, 0.496, and 0.324 log(8-OHdG/Cr) ng/mg, respectively, after adjusting for BMI and age.

The association between PAH metabolites and lipid profiles among the elderly participants was summarized in Table 4. This table shows that 1-OHNap (−0.020, 95% CI: −0.035 to −0.005) and 1-OHPyr (−0.034, 95% CI: −0.061 to −0.006) exhibited negative correlations with Apo A1, while 1-OHPyr (0.087, 95% CI: 0.015 to 0.159) and 2-OHBcPhe (0.060, 95% CI: 0.000 to 0.119) displayed positive associations with LDL-C and Apo B, respectively. However, no significant relationship was found between 8-OHdG and lipid profiles, as shown in Table 5.

Mediating effects were analyzed to further investigate the significant effects of PAH metabolites on blood lipid levels noted in Table 4. As shown in Table 6, the mediating effect of 8-OHdG on the relationship between PAH metabolites and lipid profiles did not achieve statistical significance. Nonetheless, indirect effects (denoted as “c”, as shown in Figure 2) of OH-PAHs on blood lipids were statistically significant for 1-OHNap concerning Apo A1 (−0.025, 95% CI: −0.041 to −0.009), 1-OHPyr concerning LDL-C (0.107, 95% CI: 0.011 to 0.203), and 2-OHBcPhe concerning Apo B (0.070, 95% CI: 0.005 to 0.135).

## 4. Discussions

Numerous studies have established a correlation between polycyclic aromatic hydrocarbon (PAH) metabolites and 8-hydroxydeoxyguanosine (8-OHdG) levels in urine [9,10,18]. However, to date, no research has been reported on the potential impact of urinary PAH metabolites on lipid profiles. Furthermore, the interrelationship among PAH metabolites, 8-OHdG, and blood lipid levels has not been thoroughly explored. The present study aims to investigate whether PAH metabolites induce changes in blood lipid profiles by modulating oxidative stress pathways.

The study population comprised 109 elderly individuals over 60, who may be more susceptible to environmental pollutants. The participants’ median body mass index (BMI) was 25.03 (standard deviation: 3.22). According to the World Health Organization’s BMI classification, approximately half of the subjects fell into the pre-obese category, with men showing a slightly higher prevalence than women [27]. This suggests an elevated risk of hyperlipidemia among the participants. Notably, the rates of smoking and alcohol status were significantly higher among men compared to women; about half of the male participants reported regular smoking and drinking, whereas these behaviors were observed in less than one-fifth of women. Smoking and alcohol consumption are recognized risk factors for dyslipidemia [28,29].

Urinary PAH metabolites primarily derive from exposure to PAHs through two main routes: dietary intake and inhalation [30,31]. Nutritional sources of PAHs include meat, grains, fruits, milk, and vegetables, with meat and grains being the predominant contributors [31]. Inhaled PAHs can enter the systemic circulation through respiratory exposure [32]. Several studies have indicated that dietary exposure to PAHs generally exceeds inhalation exposure in the general population [30,32,33]. Thus, managing dietary intake may reduce the concentration of PAH metabolites in the body.

In this study, it was observed that the median concentrations of PAH metabolites in urine ranged from approximately 1 to 10 μmol/mol creatinine (Cr). The low-ring PAHs, naphthalene and fluoranthene, were the primary components, accounting for 56% and 19% of the total PAH metabolites, respectively. The urinary concentrations of hydroxylated PAHs (OH-PAHs) are an integrated measure of human exposure to PAHs [23]. Previous investigations have demonstrated that urinary PAH metabolite levels are influenced by various factors, including geographical location, age, and ethnicity [23,34]. Variability in susceptibility, inhalation, and metabolism rates among different populations leads to diverse final concentrations of metabolites. The measurement of PAH metabolites is often adjusted for creatinine levels, as normal daily urinary creatinine excretion remains relatively stable and is not significantly affected by dietary protein intake or urine output. Therefore, creatinine-corrected values provide a reliable estimate of PAH metabolite concentrations. For instance, a study of college students in Guangzhou reported urinary PAH metabolite levels of 1–4 μmol/mol Cr for non-smokers and 1–9 μmol/mol Cr for smokers [35]. The median concentrations for coke oven workers in China were approximately 1–3 μmol/mol Cr [36]. A study of Portuguese firefighters indicated urinary concentrations of OH-PAHs around 1 μmol/mol Cr, with low-ring metabolites, 1-OHNap, and 1-hydroxyacenaphthene (1-OHAce), comprising 66–96% of total OH-PAHs [37]. Additionally, research across several Asian countries revealed that urinary 1-OHPyr levels in Malaysia, Korea, and Japan were comparable to those reported in North America and Western Europe. In the studies of undeveloped Asian countries, the urinary PAH metabolites in China, India, and Vietnam were 4–10 times higher than those in North America and Western Europe [34]. 

Regarding lipid profiles, Apolipoprotein A1 (Apo A1) is the principal protein component of HDL, while apolipoprotein B (Apo B) is the predominant protein in LDL and intermediate-density lipoproteins. These lipid profiles are closely associated with the risk of cardiovascular disease [38,39]. According to the 2016 Chinese guidelines for managing dyslipidemia in adults, the study population exhibited lipid and apolipoprotein levels within the normal range. The median concentrations of TC, HDL-C, LDL-C, Apo A1, and Apo B were higher in women than men. However, no significant differences in lipid levels were observed based on smoking or alcohol consumption status. 8-Hydroxy-2′-deoxyguanosine (8-OHdG) is widely recognized as a marker of oxidative stress and DNA damage [40,41]. Elevated levels of 8-OHdG often indicate an increased risk to individual health. Oxidative damage is prevalent in the body and primarily affects biological macromolecules’ structural and functional integrity, ultimately leading to gene mutations, cellular carcinogenesis, and aging. A case–control study reported that the urinary 8-OHdG concentration in the exposure group (exposure to traffic-related air pollution) was approximately 3.5 μg/g creatinine, compared to 2.5 μg/g creatinine in the control group [11]. Furthermore, urinary levels of 8-OHdG were found to be approximately 12.90 μg/g creatinine in smokers and 8.93 μg/g creatinine in non-smokers, with no statistically significant difference observed based on smoking status [35]. In summary, the concentrations of lipid profiles and 8-OHdG in our study population were moderate (Table 2).

Notably, a relationship exists between modified lipid profiles and OH-PAHs within the group of smokers. A plausible rationale for this is that smoking may exert a more significant influence on the modification of lipid profiles compared to other external PAHs, potentially obscuring the impacts of PAHs. However, the combined effects of PAHs and environmental tobacco smoke on altered lipid profiles still need to be studied. Additional research is necessary to investigate the interactions between PAHs and cigarette smoking and cardiovascular health [42].

Numerous studies have established an association between PAH metabolites and 8-OHdG [18,35,43]. A case study involving college students in Guangzhou revealed that the total concentrations of hydroxylated PAHs (∑OH-PAHs) exhibited a dose-dependent relationship with 8-OHdG levels in both smokers (r = 0.804, *p* < 0.01) and non-smokers (r = 0.493, *p* < 0.01) [35]. Additionally, a dose-dependent correlation was identified between urinary OH-PAHs and urinary 8-OHdG (*p* < 0.05 for 12 types of OH-PAHs) in a general population in China [43]. In pregnant women during their third trimester, an interquartile range increase in 2-OHNap was associated with a 14% increase in 8-OHdG levels (95% confidence interval: 0.59, 30.1) [44]. In our study, linear regression analyses indicated that increases in the logarithmic concentrations of 2-OHNap, 9-OHFlu, 3-OHFlu, 2-OHFlu, 1-OHPyr, and 6-OHChr were associated with increases in log-transformed 8-OHdG/creatinine (Cr) levels of 0.305, 0.420, 0.456, 0.467, 0.496, and 0.324 ng/mg, respectively, after adjusting for body mass index (BMI) and age. The knowledge of the mechanisms by which PAH metabolites influence 8-OHdG levels remains limited; however, one study proposed that microRNAs and their interactions with environmental factors may mediate the effects of PAH exposure on oxidative damage [45]. The evidence supports a consistent positive correlation between PAH metabolites and 8-OHdG levels.

Lipid abnormalities significantly increase the risk of cardiovascular diseases [46]. Our study suggests that urinary PAH metabolites may induce changes in blood lipid profiles. Specifically, 1-OHNap and 1-OHPyr were found to have a negative correlation with apolipoprotein A1 (Apo A1). At the same time, 1-OHPyr demonstrated a positive relationship with low-density lipoprotein cholesterol (LDL-C), and 2-OHBcPhe was positively associated with Apo B. Elevated plasma levels of Apo A1 and high-density lipoprotein cholesterol (HDL-C) are recognized as important protective factors against atherosclerosis and coronary heart disease [15,16]. Conversely, Apo B and LDL-C increases are considered risk factors for cardiovascular disease [17]. In our study, urinary PAH metabolites, identified as harmful substances, were associated with a decrease in Apo A1 and an increase in Apo B and LDL-C, suggesting that PAH metabolites may elevate the risk of cardiovascular disease. The systematic evaluation of persistent pollutants showed a correlation between elevated triglycerides and lower HDL-C with higher fat-soluble contaminants such as polychlorinated biphenyls and dibenzofurans [47]. To date, no research has directly assessed the effects of PAH metabolites on blood lipids. However, some studies have focused on the association between lipid peroxidation (primarily measured by malondialdehyde, MDA) and PAH levels, providing evidence that lipid peroxidation is linked to PAH metabolite concentrations [19,45,48].

Consequently, we aimed to investigate whether PAHs influence blood lipids through oxidative stress pathways. We did not find any significant relationship between 8-OHdG levels and lipid profiles. A cross-sectional study in Australia identified a negative correlation between 8-OHdG and HDL-C [49]. Additionally, a study involving type II diabetic patients reported a weak correlation between 8-OHdG levels and triglycerides (r = 0.230, *p* = 0.074), while urinary 8-OHdG levels showed no correlation with total cholesterol [50]. Oxidative stress in the body can cause an increase in reactive oxygen species, which may further affect blood lipids. 

In addition, our study investigated the mediating effect of oxidative stress. Mediation analysis has been widely utilized across various disciplines, effectively identifying the presence and extent of the mediation effects [26,51,52]. However, our mediation analysis did not reveal a mediating effect of 8-OHdG in this study. Nevertheless, we found statistically significant indirect effects (c’) of PAH metabolites on blood lipids, specifically, 1-OHNap was associated with Apo A1 (−0.025, 95% CI: −0.041, −0.009), 1-OHPyr with LDL-C (0.107, 95% CI: 0.011, 0.203), and 2-OHBcPhe with Apo B (0.070, 95% CI: 0.005, 0.135). These findings suggest that 8-OHdG does not serve as an intermediary in the relationship between PAH metabolites and lipid profiles, indicating the presence of alternative pathways influencing this process. Furthermore, alterations in lipid profiles may also be linked to factors such as hormones, enzymes, and genetic defects. Thus, the mechanisms by which PAH metabolites affect lipid profiles warrant further investigation.

Several limitations of this study should be acknowledged. First, the research design was a cross-sectional survey with a small sample size, which may lead to false-negative results regarding the correlation between oxidative stress and blood lipids. Second, lipid peroxidation markers (such as MDA) were not measured, which potentially serve as intermediate products linking 8-OHdG to blood lipids. The evidence suggests that urinary PAH metabolites can influence oxidative stress and lipid peroxidation. Third, other co-existing air pollutants, such as NO_2_ and O_3_, may also cause oxidative damage. However, we did not monitor these pollutants. Therefore, our study is a preliminary investigation to encourage further research.

## 5. Conclusions

This study indicated that increased urinary PAH metabolites are associated with elevated levels of urinary 8-OHdG and alterations in blood lipid profiles. Although we did not observe a direct relationship between 8-OHdG and lipid profiles through mediation analysis, the PAH metabolites may induce changes in lipid levels through pathways other than oxidative stress.

## Figures and Tables

**Table 1:** Statistical characteristics of the baseline data.

Variable	Male (n = 46)	Female (n = 63)	Total (n = 109)	F	*p*
Age (mean ± SD)	69 ± 7.0	67 ± 5.0	68 ± 6.0	1.73	0.87
BMI (kg/m^2^) (mean ± SD)	25.61 ± 3.34	24.60 ± 3.09	25.03 ± 3.22	1.62	0.11
Smoking status	54%	16%	32%	18.05	<0.01
Alcohol status	44%	5%	21%	23.94	<0.01

**Table 2:** Concentrations of the lipid profiles and 8-OHdG among elderly individuals of different genders.

Variables	Median (P_25_, P_75_)			Z	*p*
	Male (n = 46)	Female (n = 63)	Total (n = 109)		
TC (mmol/L)	5.07 (4.54, 5.71)	5.61 (5.17, 6.26)	5.42 (4.86, 5.95)	−2.88	0.004
TG (mmol/L)	1.31 (0.92, 1.94)	1.44 (1.18, 1.85)	1.41 (1.03, 1.90)	−1.13	0.260
HDL-C (mmol/L)	1.14 (1.03, 1.31)	1.31 (1.17, 1.48)	1.26 (1.09, 1.43)	−2.87	0.004
LDL-C (mmol/L)	3.06 (2.63, 3.57)	3.41 (2.90, 3.79)	3.24 (2.74, 3.64)	−2.11	0.035
Apo A1(g/L)	1.24 (1.17, 1.29)	1.32 (1.26, 1.39)	1.29 (1.21, 1.36)	−3.87	<0.001
Apo B (g/L)	0.89 (0.77, 0.98)	0.94 (0.88, 1.05)	0.92 (0.81, 1.04)	−2.02	0.043
Apo B/Apo A1	0.72 (0.61, 0.83)	0.72 (0.65, 0.81)	0.72 (0.62, 0.82)	−0.09	0.929
8-OHdG (ng/mg Cr)	4.98 (3.20, 7.17)	5.64 (3.15, 8.92)	5.44 (3.15, 8.48)	−0.92	0.357

**Table 3:** The association between the 8-OHdG and OH-PAHs (×10^−2^).

lg (8OHdG)	lg (1-OHNap)	lg (2-OHNap)	lg (9-OHFlu)	lg (3-OHFlu)	lg (2-OHFlu)	lg (1-OHPyr)	lg (2-OHBcPhe)	lg (6-OHChr)
Model 1 ^#^	16.8 * (1.8–31.8)	30.9 * (14.0–47.8)	43.4 * (25.3–61.4)	47.5 * (31.0–63.9)	48.0 * (29.5–66.4)	51.4 * (28.5–74.3)	9.7 (−12.3–31.8)	33.9 * (15.0–52.7)
Model 2 ^&^	14.4 (−1.0–29.9)	30.5 * (13.1–47.9)	42.0 * (23.3–60.7)	45.6 * (28.5–52.7)	46.7 * (27.6–65.8)	49.6 * (26.0–73.2)	8.4 (−13.9–30.6)	32.4 * (12.9–51.9)

* denotes *p* < 0.05; ^#^ Model 1: base model without any adjustment. ^&^ Model 2: adjusted by age and BMI.

**Table 4:** The association between the log-transformed PAH metabolites and lipid profiles (×10^−2^).

lg(8OHdG)	lg(1-OHNap)	lg(2-OHNap)	lg(9-OHFlu)	lg(3-OHFlu)	lg(2-OHFlu)	lg(1-OHPyr)	lg(2-OHBcPhe)	lg(6-OHChr)
lg(TC) (*10^−2^)								
Model 1 ^#^	−0.7 (−3.9–2.5)	0.1 (−3.8–4.0)	2.7 (−1.8–7.2)	1.6 (−2.6–5.7)	3.7 (−1.2–8.5)	2.9 (−2.8–8.5)	2.7 (−3.0–8.5)	2.8 (−2.6–8.2)
Model 2 ^&^	−0.9 (−4.1–2.4)	0.3 (−3.8–4.3)	2.8 (−1.8–7.5)	1.3 (−3.0–5.6)	3.2 (−1.8–8.2)	3.9 (−1.9–9.7)	2.8 (−3.3–8.9)	2.7 (−2.8–8.3)
lg(TG)								
Model 1	−4.2 (−13.4–5.0)	−2.5 (−14.6–9.6)	9.1 (−3.3–21.4)	6.1 (−6.1–18.4)	0.10.3 (−3.0–23.7)	8.1 (−8.0–24.3)	5.3 (−11.3–21.8)	8.3 (−7.7–24.2)
Model 2	−3.2 (−12.6–6.1)	−1.2 (−13.6–11.2)	11.8 (−0.9–24.5)	8.7 (−3.9–21.3)	12.4 (−1.2–26.1)	11.2 (−5.2–27.7)	4.1 (−13.1–21.3)	11.7 (−4.4–27.9)
lg(HDL-C) (*10^−2^)								
Model 1	−0.1 (−5.3–5.0)	1.5 (−5.3–8.3)	−1.7 (−8.5–5.0)	−1.8 (−8.6–5.0)	−1.5 (−8.8–5.8)	−1.0 (−10.4–8.4)	0.0 (−8.7–8.6)	−2.0 (−10.7–6.6)
Model 2	−1.4 (−6.3–3.5)	0.3 (−6.4–7.0)	−4.2 (−10.9–2.6)	−4.1 (−10.8–2.6)	−3.8 (−10.9–3.3)	−2.7 (−12.2–6.7)	0.2 (−8.2–8.7)	−4.9 (−13.1–3.3)
lg(LDL-C) (*10^−2^)								
Model 1	1.0 (−3.2–5.2)	0.3 (−4.9–5.5)	4.9 (−1.2–10.9)	3.3 (−2.1–8.8)	6.0 (−0.6–12.6)	6.2 (−1.1–13.5)	5.0 (−2.6–12.5)	4.3 (−2.8–11.5)
Model 2	1.4 (−2.9–5.7)	1.2 (−4.0–6.4)	6.1 (−0.1–12.3)	3.9 (−1.7–9.5)	6.3 (−0.4–12.9)	8.7 * (1.5–15.9)	5.4 (−2.4–13.2)	5.2 (−2.0–12.4)
lg(Apo A1) (*10^−2^)								
Model 1	−1.7* (−3.2–−0.2)	−1.2 (−3.3–0.8)	−0.3 (−2.7–2.1)	−0.6 (−2.7–1.5)	−0.9 (−3.2–1.4)	−2.8 * (−5.5–−0.1)	−0.7 (−3.4–2.0)	−0.7 (−3.3–1.8)
Model 2	−2.0* (−3.5–−0.5)	−1.7 (−3.8–0.3)	−1.0 (−30.5–1.4)	−1.2 (−3.3–0.9)	−1.6 (−3.8–0.7)	−3.4 * (−6.1–−0.6)	−0.9 (−3.6–1.9)	−1.3 (−3.9–1.2)
lg(Apo B) (*10^−2^)								
Model 1	1.0 (−2.4–4.4)	−2.3 (−6.4–1.8)	−0.5 (−5.4–4.4)	−2.1 (−6.5–2.3)	0.8 (−4.5–6.1)	1.9 (−4.3–8.0)	6.2 * (0.5–11.9)	−1.9 (−7.7–3.9)
Model 2	1.1 (−2.4–4.6)	−2.2 (−6.4–2.1)	−0.4 (−5.4–4.7)	−2.2 (−6.8–2.4)	0.5 (−4.9–5.9)	2.8 (−3.6–9.2)	6.0 * (0.0–11.9)	−1.4 (−7.4–4.6)
lg(Apo B/Apo A1) (*10^−2^)								
Model 1	2.7 (−1.3–6.6)	−1.1 (−6.0–3.8)	−0.2 (−5.6–5.2)	−1.5 (−6.6–3.7)	1.7 (−4.2–7.6)	4.7 (−2.5–11.9)	6.9 * (0.4–13.3)	−1.2 (−7.9–5.6)
Model 2	3.1 (−0.9–7.1)	−0.4 (−5.4–4.5)	0.6 (−5.0–6.3)	−1.0 (−6.3–4.3)	2.1 (−3.9–8.0)	6.2 (−1.3–13.6)	6.9 * (0.2–13.6)	0.0 (−6.8–6.8)

* denotes *p* < 0.05. ^#^ Model 1: base model without any adjustment. ^&^ Model 2: adjusted by age and BMI.

**Table 5:** The association between 8-OHdG and lipid profiles of the elderly (×10^−2^).

lg(8OHdG)	lg(TC)	lg(TG)	lg(HDL-C)	lg(LDL-C)	lg(Apo A1)	lg(Apo B)	lg(Apo B/Apo A1)
Model 1 ^#^	1.1 (−4.4–6.6)	3.1 (−11.1–17.4)	1.0 (−6.7–8.7)	1.8 (−5.6–9.1)	−0.3 (−3.0–2.4)	−3.5 (−9.3–2.2)	−3.2 (−9.6–3.1)
Model 2 ^&^	1.0 (−4.7–6.7)	5.9 (−8.5-.20.3)	−1.4 (−8.9–6.2)	2.6 (−5.0–10.1)	−0.9 (−3.6–1.8)	−3.2 (−9.1–2.7)	0.4 (−0.2–1.1)

^#^ Model 1: base model without any adjustment. ^&^ Model 2: adjusted by age and BMI.

**Table 6:** The mediation effect of 8-OHdG on PAH metabolites and lipid profile adjusted by age and BMI (×10^−2^).

Mediating Process (8-OHdG)	a ^1^	b ^2^	c ^3^	c’ ^4^	a × b
1-OHNap-Apo A1	14.4 (−1.0, 29.9)	0.4 (−2.0, 2.8)	−2.4 * (−4.0, −0.9)	−2.5 * (−4.1, −0.9)	0.1 (−0.3, 0.6)
1-OHPyr-LDL-C	49.6 * (26.0, 73.2)	−2.1 (−12.2, 8.0)	9.6 * (1.5, 17.8)	10.7 * (1.1, 20.3)	−1.0 (−5.6, 3.7)
1-OHPyr-Apo A1	49.6 * (26.0, 73.2)	−0.1 (−3.9, 3.8)	−3.1 (−6.1, 0.1)	−3.1 (−6.7, 0.6)	−0.1 (−1.7, 2.3)
2-OHBcPhe-Apo B	8.4 (−13.9, 30.6)	−10.2 * (−19.4, −1.1)	6;1 (−0.6, 12.9)	7.0 * (0.5, 13.5)	−0.9 (−3.1, 1.2)

* denotes *p* < 0.05. ^1^ a denotes the effects of OH-PAHs on 8-OHdG. ^2^ b denotes the effects of 8-OHdG on lipid profiles. ^3^ c denotes the total effect of OH-PAHs on lipid profiles. ^4^ c’ denotes the direct effect of OH-PAHs on lipid profiles after controlling for 8-OHdG.

## Data Availability

The data presented in this study are available on request from the corresponding author.

## Supplementary Materials

The following supporting information can be downloaded at: https://www.mdpi.com/article/10.3390/toxics12100748/s1, Text S1: Analysis Procedures of OH-PAHs; Figure S1: Analysis of PAH metabolites in urine samples by GC/MS; Table S1: Instrumental parameters of GC-MS; Table S2: Concentrations of lipid profiles and 8-OHdG in the different smoking statuses of the elderly; Table S3: Concentrations of lipid profiles and 8-OHdG in different alcohol status of the elderly.
