# Supplementary material for: The Effect of Urinary Polycyclic Aromatic Hydrocarbon Metabolites on Lipid Profiles: Does Oxidative Stress Play a Crucial Mediation Role?
Source: Toxics. 2024 Oct 15;12(10):748. doi: 10.3390/toxics12100748 (PMC11511148; doi:10.3390/toxics12100748)

toxics

Supplementary Information

The Effect of Urinary Polycyclic Aromatic Hydrocarbon Metabolites on Lipid Profiles: Does oxidative stress play a crucial mediation role?

Yuting Wang[1#], Jia Xu[2#], Liujie Yang[1], Nan Zhang[2], Liwen Zhang[1*], Bin Han[2*]

1. Department of Occupational and Environmental Health, School of Public Health, Tianjin Medical University, Tianjin, 300070, China
2. State Key Laboratory of Environmental Criteria and Risk Assessment, Chinese Research Academy of Environmental Sciences, Beijing, 100012, China.

# These two authors contributed equally to this paper.

*   Correspondence: Bin Han (hanbin@craes.org.cn) and Liwen Zhang (zhangliwen@tmu.edu.cn)

Text S1 Analysis Procedures of OH-PAHs

(1) Preparation of Internal Standard Solution (250 ng/mL):

A total of 50 μL each of the 50 g/mL standard solutions of anthracene-D10 (Dr. Ehrenstorfer GmbH, Germany) and pyrene-D10 (Anpel Laboratory Technologies Inc., China) were pipetted into a 10 mL brown volumetric flask. The flask was filled to the mark with isooctane and mixed thoroughly to obtain a 250 ng/mL internal standard solution, which was then stored in the dark at -20°C for future use.

(2) Preparation of Polycyclic Aromatic Hydrocarbon Metabolite Mixed Standard Solution (50 ng/mL):

A total of 100 μL each of the 10 μg/mL standard solutions of 1-hydroxynaphthalene (1-OHNap), 2-hydroxynaphthalene (2-OHNap), 2-hydroxyfluorene (2-OHFlu), 3-hydroxyfluorene (3-OHFlu), 1-hydroxyphenanthrene (1-OHPhe), 2-hydroxyphenanthrene (2-OHPhe), 4-hydroxyphenanthrene (4-OHPhe), 3+9-hydroxyphenanthrene (3-OHPhe + 9-OHPhe), 1-hydroxypyrene (1-OHPyr), 6-hydroxychrysene (6-OHChr), and 2-hydroxy-dibenzofuran (2-OHDBF) (AccuStandard, USA) were pipetted into a 5 mL brown volumetric flask. The flask was filled to the mark with isooctane and mixed thoroughly to obtain a 50 ng/mL polycyclic aromatic hydrocarbon metabolite mixed standard solution, which was then stored in the dark at -20°C for future use.

(3) Preparation of Recovery Marker Solution (250 ng/mL):

A total of 50 μL of the 50 μg/mL standard solution of [2H9]-1-hydroxypyrene ([2H9]-1-OHPyr) (Shanghai ZZBIO Co., LTD, China) was pipetted into a 10 mL brown volumetric flask. The flask was filled to the mark with methanol and mixed thoroughly to obtain a 250 ng/mL recovery marker solution, which was then stored in the dark at -20°C for future use.

(4) Sample Preparation of Urine:

The urine sample was taken out from the -80°C freezer and allowed to thaw completely at room temperature.

(5) Enzymatic Hydrolysis:

A pipette transferred 1.5 mL of the urine sample into a 12 mL screw-cap glass tube. Subsequently, 1 mL of sodium acetate buffer (pH = 5.5) was added, followed by sequential addition of 50 μL of diluted hydrochloric acid (0.5 M), 50 μL of recovery marker solution, and 35 μL of β-glucuronidase using a piston-driven pipette. The tube was capped, inverted several times to mix, and placed in a thermostatic shaking water bath at 37°C for 12 hours in the dark.

(6) Liquid-Liquid Extraction:

2.5 mL of organic extraction solvent was added to the enzymatically hydrolyzed urine sample using a pipette. The mixture was vortexed and shaken on a horizontal shaker for 10 minutes. After shaking, the sample was centrifuged at 3800 rpm for 10 minutes, followed by ultrasonic treatment for 3 minutes. The sample was centrifuged again at 3800 rpm for 10 minutes, and the supernatant from the second centrifugation was collected. This extraction step was repeated three times, and all supernatants were combined.

(7) Derivatization:

The supernatant collected from step (2) was concentrated near dryness under a nitrogen stream. To the residue, 125 μL of methanol, 50 μL of toluene, and 50 μL of trimethylsilyl diazomethane were added, and the mixture was placed in a thermostatic shaking water bath at 30°C for 1 hour in the dark for derivatization. The derivatized liquid was then concentrated to near dryness again, and 100 μL of dichloromethane was added to obtain the concentrated sample.

(8) Solid-Phase Extraction:

A 12 mL column of activated SiO2 SPE was washed with n-hexane. Once the n-hexane had drained to the silica layer, approximately 100 µL of the concentrated sample from step (3) was applied to the SPE column. The sample container was washed with about 3 mL of n-hexane in three portions, and the washings were combined and applied to the column. After loading, the column was further washed with n-hexane while the eluent was collected in an 8 mL screw-cap glass tube. The sample was then eluted with dichloromethane, followed by a mixture of dichloromethane and n-hexane, collecting the elution in two separate 8 mL tubes.

(9) Nitrogen Blowdown and Volume Adjustment:

The eluent obtained from step (4) was concentrated to near dryness under nitrogen. To this, 100 µL of isooctane was added. After vortex mixing, all the liquid was transferred to a brown injection vial equipped with an internal pipette. Finally, 50 µL of the internal standard solution was added, and the sample was stored in the dark at -20°C until analysis.

(10) Instrumental analysis

Gas chromatography-triple quadrupole mass spectrometry determined the concentration of PAH metabolites in urine. The parameters of the instrument are shown in Table S1.

To quantify the OH_PAHs, we used a one-point calibration method. The prepared mixed standard solution (20.0 ng/mL) was used as the internal standard. The area ratio of the quantitative ion peaks of the target compound to the internal standard compound was calculated to obtain the concentration of the target compound.

Table S1 Instrumental parameters of GC-MS

GC	Parameters and conditions	
chromatographic column	DB-5 ms； 15 m； 0.25 mm×0.25 μm；	
column temperature	70℃	
Inlet temperature	280℃	
Injection method and volume	unsplit stream Injection, 1μL	
carrier gas	high purity helium (99.9%)	
temperature programming	110℃ for 1min; raised at 5 ℃/min to 200 ℃ and maintained for 10 min; raised to 200 ºC at 20 ºC/min; raised at 5 ℃/min to 300 ℃ and maintained for 5 min	
**MS**	**Parameters and conditions**	
Ion source	EI source (70eV)	
Ion source temperature	230℃	
Interface temperature	280℃	
Monitoring mode	MRM	
Scanning charge-mass ratio of PAHs metabolites	1-OHNap	m/z 158 and m/z 115
	2-OHNap	
	2-OHFlu	m/z 196 and m/z 152
	3-OHFlu	m/z 196 and m/z 181
	1-OHPhe	m/z 208 and m/z 165
	2-OHPhe	
	4-OHPhe	
	3+9-OHPhe	
	1-OHPyr	m/z 217 and m/z 189
	6-OHChr	m/z 258 and m/z 215
	2-OHDBF	m/z 198 and m/z 183

Table S2 Concentrations of lipid profiles and 8-OHdG in the different smoking statuses of the elderly

Variables	$M(P_{25},P_{75})$			Z	*P*
	Smoking（n=35）	No smoking（n=74）	Total（n=109）		
TC (mmol/L)	5.31(4.93,5.84)	5.52(4.85,6.08)	5.42(4.86,5.95)	-0.62	0.533
TG (mmol/L)	1.35(0.93,1,73)	1.44(1.09,1.93)	1.41(1.03,1.90)	-0.81	0.417
HDL-C (mmol/L)	1.29(1.08,1.44)	1.25(1.10,1.42)	1.26(1.09,1.43)	-0.36	0.721
LDL-C (mmol/L)	3.13(2.74,3.56)	3.36(2.74,3.67)	3.24(2.74,3.64)	-0.90	0.367
Apo A1(g/L)	1.28(1.19,1.33)	1.29(1.21,1.36)	1.29(1.21,1.36)	-0.78	0.438
Apo B (g/L)	0.91(0.81,1.00)	0.92(0.81,1.04)	0.92(0.81,1.04)	-0.38	0704
Apo B/Apo A1	0.72(0.62,0.81)	0.72(0.64,9.81)	0.72(0.62,0.82)	-0.06	0.951
8-OHdG(ng/mg)	5.04(3.04,7.36)n=26	5.54(3.19,8.48)n=59	5.44(3.15,8.48)	-0.76	0.445

Table S3 Concentrations of lipid profiles and 8-OHdG in different alcohol status of the elderly

Variables	$M(P_{25},P_{75})$			Z	*P*
	Alcohol（n=23）	No alcohol（n=86）	Total（n=109）		
TC (mmol/L)	5.26(4.93,5.83)	5.45(4.85,6.08)	5.42(4.86,5.95)	-0.64	0.525
TG (mmol/L)	1.54(1.24,2.07)	1.35(1.00,1.82)	1.41(1.03,1.90)	-1.17	0.242
HDL-C (mmol/L)	1.18(1.02,1.31)	1.28(1.11,1.45)	1.26(1.09,1.43)	-1.56	0.120
LDL-C (mmol/L)	3.24(2.74,3.57)	3.27(2.74,3.64)	3.24(2.74,3.64)	-0.54	0.590
Apo A1(g/L)	1.27(1.21,1.29)	1.30(1.21,1.37)	1.29(1.21,1.36)	-1.62	0.105
Apo B (g/L)	0.91(0.82,0.98)	0.92(0.80,1.04)	0.92(0.81,1.04)	-0.33	0.744
Apo B/Apo A1	0.72(0.65,0.81)	0.71(0.61,0.82)	0.72(0.62,0.82)	-0.56	0.575
8-OHdG(ng/mg)	3.72(2.34,6.28)n=19	5.55(3.32,8.52)n=66	5.44(3.15,8.48)	-1.37	0.170

Figure S1. Analysis of PAH metabolites in urine samples by GC/MS

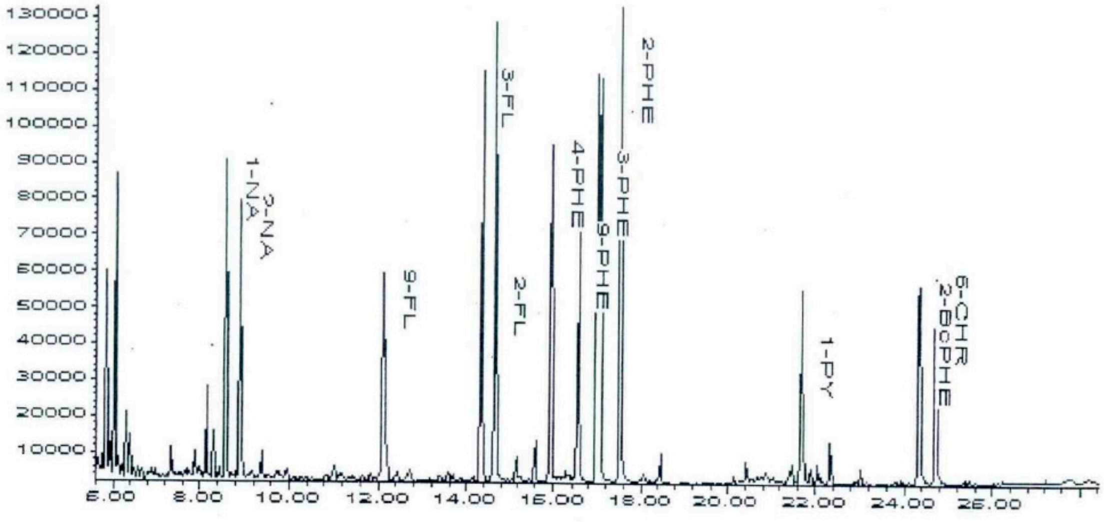

Supplement: Supplementary file 1 [file toxics-12-00748-s001.zip › toxics-3168199-supplementary.pdf]
